# Microbiological Safety of Ornamental Edible Flowers

**DOI:** 10.3390/foods15152697

**Published:** 2026-07-30

**Authors:** Ivan Ciliberti, Assunta De Lella, Anna Ardévol, Sanja Ćavar Zeljković, Francesco De Marco, Francesco Corvino, Lucia De Luca, Maria Aponte, Raffaele Romano

**Affiliations:** 1 Department of Agricultural Sciences, University of Naples Federico II, 80055 Portici, Italy; ivan.ciliberti@unina.it (I.C.); francesco.demarco@unina.it (F.D.M.); francesco.corvino@unina.it (F.C.); lucia.deluca@unina.it (L.D.L.); rafroman@unina.it (R.R.); 2 Istituto Zooprofilattico Sperimentale del Mezzogiorno (IZS), 80055 Portici, Italy; assunta.delella@izsmportici.it; 3 Department of Biochemistry and Biotechnology, Molecular Bioactivity of Foods (MoBioFood), Universitat Rovira i Virgili, 43007 Tarragona, Spain; anna.ardevol@urv.cat; 4 Czech Agrifood Research Center (CARC), Šlechtitelů 29, 78371 Olomouc, Czech Republic; sanja.cavar@upol.cz; 5 Czech Advanced Technology and Research Institute (CATRIN), Palacký University Olomouc, Šlechtitelů 27, 78371 Olomouc, Czech Republic

**Keywords:** ornamental edible flowers, *Viola* × *wittrockiana*, *Antirrhinum majus*, microbial colonisation, pathogens, HTS

## Abstract

The present work focuses on the microbial characterisation of edible flowers intended solely for aesthetic purposes. This market segment is especially critical. In fact, due to the limited resilience to mechanical shocks, edible flowers are rather difficult to wash. All analysed samples exhibited substantial microbial loads, dominated by *Enterobacteriaceae*, *Pseudomonadaceae*, coliforms, yeasts, and moulds. Refrigerated storage (5 days at 4 °C) resulted in a marked and consistent increase in microbial populations, as confirmed by multivariate analysis, which identified storage as the primary driver of microbial dynamics. Primary foodborne pathogens were not detected; however, several bacterial species with opportunistic pathogenic potential, including *Pseudomonas juntendi*, *Enterobacter asburiae*, *Ralstonia mannitolilytica*, and *Pantoea agglomerans*, were identified in pansies, while *Pseudomonas aeruginosa* was detected in snapdragon. Multivariate analyses further revealed that in addition to storage, flower type and production source contributed to variability in microbial composition. Moreover, colour-based analysis indicated that corolla colour may influence microbial profiles, with differences becoming more pronounced after storage in pansies and already detectable at purchase in snapdragons. Overall, these findings demonstrate that ornamental edible flowers can have high and dynamic microbial populations, with storage conditions representing the dominant factor influencing microbial proliferation.

## 1. Introduction

Throughout history, flowers have been used for both decoration and culinary purposes [1]. The use of flowers in culinary practices dates back to ancient civilisations [2]. Romans were known to consume flowers and inflorescences (Floriphagia) such as lavender, violets, and roses [1].

Consumers are increasingly interested in edible flowers due to their ability to enhance the aesthetic appeal of foods while also providing bioactive compounds and micronutrients [3]. A variety of flowers have been described as edible decorations for desserts and cakes or as ingredients in salads [4]. According to currently available sources, edible flowers comprise 180 species across 100 genera and 97 botanical families [5]. They also contribute significantly to regional culinary identity and gastronomic innovation [4].

Several studies have been conducted in recent years, particularly on the antioxidant potential of edible flowers [6], highlighting their content of carotenoids, vitamins (A, C, E), and minerals [7]. Edible flowers are rich in phytochemicals with antioxidant activity, including flavonoids, phenolic acids, lignans, and tannins [8].

On the other hand, the microbiological component and safety of edible flowers remain poorly characterised. Edible flowers should be regarded as extremely perishable [2] as they have high moisture content and nutrient availability that favour microbial growth and enzymatic activity [9]. Indeed, floral tissues such as pollen, nectar, and petals provide a nutrient-rich environment supporting microbial colonisation [8].

The term “edible flower” lacks a precise meaning without proper context. It is generally used as a collective term referring to flowers considered suitable for human consumption or food contact [10]. Edible flowers are generally classified into three categories: vegetable flowers, fruit flowers, and medicinal flowers. Flowers such as broccoli, cauliflower, artichokes, and capers, which are commonly used on a day-to-day basis, are treated as traditional vegetables by consumers and are eaten after being washed and cooked. Conversely, ornamental flowers are added for scent, and above all, for the visual appearance they can give to ready dishes, drinks, or desserts. Importantly, ornamental flowers are often consumed raw without prior washing or processing [11].

Up until now, no international organisation, such as the Food and Agriculture Organisation of the United Nations (FAO), World Health Organisation (WHO), Food and Drug Administration (FDA), or European Food Safety Authority (EFSA), has released official lists of edible and non-edible flowers [12]. Nonetheless, some information regarding flower safety can be found in European Regulation (EC) No. 258/97, which deals with novel foods and novel food ingredients. Therefore, there are no legal restrictions on the commercialisation of edible flowers [12]. Nevertheless, the Rapid Alert System for Food and Feed (RASFF) reported multiple contamination events associated with edible flowers [7,13]. The primary issues are caused by bacteria (*Salmonella* spp.) or chemical compounds such as sulfites, dimethoate (an insecticide), and diethyl-meta-toluamide (an insect repellent) [14]. In the European Union, pesticide residues are regulated under Regulation (EC) No. 396/2005 [15], which establishes maximum residue levels for herbs and edible flowers. Conversely, there is no EU indication concerning microbiological aspects, despite the *Salmonella* hazard detection [11,14]. Furthermore, although microbiological safety aspects have been recently reviewed [11], available data are often aggregated across diverse flower types, including those requiring cooking before consumption, such as *Cucurbita pepo* L. [16] and *Carica papaya* L. [17], or flowers that are usually consumed as powder or as oil, just like saffron stigma (*Crocus sativus* L.) or clove flower bud (*Syzygium aromaticum*), respectively [18]. Additionally, studies frequently include plant materials used as flavouring agents that can be washed or processed before use, like *Lavandula angustifolia* Mill. [19], *Ocimum basilicum*, *Origanum vulgare*, *Salvia rosmarinus*, and *Thymus vulgaris* [20].

According to a 2026 analysis by Assofloro, Coldiretti, and three universities, the production of edible flowers in Italy is worth around 7 million euros, accounting for 20% of the European total. The main production regions are Puglia and Campania (https://www.coldiretti.it/economia/myplant-fiori-da-mangiare-nel-piatto-di-1-italiano-su-4 accessed 6 July 2026).

Given this, this study focuses on the microbial characterisation of ornamental edible flowers intended for raw consumption. This part of the flower market is especially challenging. In fact, due to the limited resilience to mechanical shocks, ornamental flowers cannot be easily washed. Furthermore, flowers are highly exposed to environmental microbiota and insect vectors, which may contribute to higher microbial contamination compared to other edible plant tissues.

Specifically, the microbial load and composition of two commonly marketed ornamental edible flowers in the EU, pansy (*Viola* × *wittrockiana*) and snapdragon (*Antirrhinum majus*), were investigated using a polyphasic approach combining conventional culture-based methods and 16S rRNA gene amplicon sequencing. The chosen flower types are largely used as garnishes in salads, soups, vinegar, drinks, and for decorating desserts; their phytochemical profiles have also been previously characterised [21].

## 2. Materials and Methods

### 2.1. Sampling and Preliminary Microbial Evaluation

Through an exploratory survey of the larger fruit and vegetable markets in the province of Naples (Campania Region, Italy), packaged edible ornamental flowers were reported to be sourced from three main producers. In all cases, mixed flowers were packaged in disposable, resealable polypropylene food containers. The number of flowers per package varied between 30 and 60 pieces. The most represented flower species were snapdragon (*Antirrhinum majus*), violet heartsease pansy (*Viola* × *wittrockiana*), violets (*Viole cornute*), sweet Williams (*Dianthus barbatus*), and common carnations (*Dianthus caryophyllus*), followed by marigold (*Tagetes patula*), ageratum (*Ageratus* spp.), vervain (*Verbena hybrida*), daisy (*Leucanthemum vulgare*), shepherd’s bourse (*Capsella bursa*-*pastoris*), and fuchsia (*Fuchsia hybrida*). Two types of packaging were labelled and are herein reported as Brand 1 and Brand 2. Brand 1 labels provided indications about the number of flowers in the box, the storage temperature (2–8 °C), and the washing requirement before use. Brand 2 labels stated that the product was not washed and provided a date but did not specify whether it referred to the harvest or packaging date. The third type of edible flower packaging had no label and is herein referred to as Anonymous (Supplementary Figure S1).

Samples (at least 3 boxes for each producer) were analysed on the date of purchase and after five days of storage at 4 °C. In detail, the sample to be analysed was collected by joining the contents of three packages. Flower samples (at least ten grams) were serially diluted and plated on Total Plate Count agar (PCA) after incubation at 30 °C for 24–48 h for the assessment of the total aerobic mesophilic microflora.

### 2.2. Microbial Characterisation of Mixed Edible Flowers

Samples of mixed flowers from the Brand 2 producer were analysed two days after the date of harvest/packaging reported on the label, and again after five days (total storage time of one week) at 4 °C. Samples to be analysed at each time were prepared by combining the contents of three packages as described in Section 2.1. Flower samples (at least ten grams) were serially diluted and plated on Total Plate Count agar (PCA) to assess the total aerobic mesophilic microflora. *Enterobacteriaceae* and coliforms were monitored on Violet Red Bile Glucose agar (VRBGA) and Violet Red Bile Lactose agar (VRBLA) after incubation at 37 °C for 24–48 h in microaerophilic conditions generated by the double-layer technique. Coagulase-positive staphylococci and *Escherichia coli* were assessed on Baird Parker added to Egg Yolk Tellurite Emulsion (BP) and Tryptone Bile X-Gluc agar (TBX) media, respectively, in both cases after incubation at 37 °C for 24–48 h. *Pseudomonadaceae* were evaluated on Pseudomonas Agar Base plus CFC supplement (PSA) after incubation at 28 °C for 48 h, and lactic acid bacteria (LAB) on De Man–Rogosa–Sharpe (MRS) after incubation at 30 °C for 48 h. Yeasts and moulds were separately counted on Dichloran-Rose Bengal Chloramphenicol Agar (DRBC) after incubation at 28 °C for 48 h.

Additionally, each sample was examined according to ISO procedures [22,23,24] to detect the presence of pathogens such as *Bacillus cereus*, *Salmonella*, and *Listeria* spp.

All media and supplements were provided by Condalab (Madrid, Spain). Analyses were performed in duplicate.

### 2.3. Snapdragon and Pansy Microbial Characterisation

Samples of snapdragon and pansy (Brand 2 and Anonymous), assembled as described in Section 2.2, were subject to the same set of analyses as previously described. Flowers were analysed at time 0 (T0), namely on the day of purchase (in the Brand 2 case, two days after the date indicated on the label) and after a further five days of storage at 4 °C (T5). Additionally, flowers were also evaluated after being split by colour: deep purple, white edged in violet, and orange/violet for pansy at both T0 and T5; red orange, pink, white, and amber for a first batch of snapdragon flowers (Brand 2), and fuchsia, yellow, deep purple, white, and vermillion for a second snapdragon batch (Anonymous) only at T0. Furthermore, at least ten colonies were randomly picked from the countable plates of PCA, PSA, and MRS (T0) to analyse the dominant microflora in pansy and snapdragon from Brand 2. In detail, single colonies were purified by repeated streaking on the same counting media. Cultures were checked for Gram reaction and catalase activity and identified by 16S rRNA sequencing using primers and PCR conditions described by Weisburg et al. [25]. DNA was extracted using the protocol described by Wang et al. [26]. PCR amplicons were purified using the QIAquick Gel Extraction Kit (Qiagen, Venlo, The Netherlands). Sequencing was carried out by Eurofins Genomics (Ebersberg, Germany).

### 2.4. High-Throughput Sequencing Analysis of Pansy and Snapdragon Bacterial Communities

The bacterial community of mixed flowers (M0 and M5), as well as pansies (P0 and P5) and snapdragons (S0 and S5), from Brand 2, which had previously been analysed by conventional counting methods as outlined in Section 2.2 and Section 2.3, respectively, was assessed. Total DNA extraction from samples was carried out using NucleoSpin Food (Macherey-Nagel, Düren, Germany). Bacterial communities were assessed by high-throughput sequencing analysis (HTS) of the amplified V3–V4 regions within the 16S rRNA gene (~460 bp). PCR was carried out using primers (S-D-Bact-0341-b-S-17/S-D Bact0785-a-A-21) connecting with barcodes [27]. PCR products with the proper size were selected by 2% agarose gel electrophoresis. The same amount of PCR products from each sample was pooled, end-repaired, A-tailed, and further ligated with Illumina adapters. The library was checked with Qubit and real-time PCR for quantification and a bioanalyser for size distribution detection. Quantified libraries were pooled and sequenced on a paired-end Illumina platform to generate 250 bp paired-end raw reads (Illumina, San Diego, CA, USA). Paired-end reads were joined using FLASH [28]. The DADA2 method [29] was used for noise reduction. ASVs (amplicon sequence variants) were further filtered using QIIME2 software (Version 221, QIIME2-202202) and identified against the Silva Database 138.1.

The 16S rRNA gene sequences are available at the Sequence Read Archive (SRA) of the National Center for Biotechnology Information (NCBI), under accession number PRJNA1444999.

### 2.5. Statistical Analysis

All analyses were performed in RStudio (R version 4.4.1). Microbial counts were expressed as Log CFU/g, and values below the detection limit (10 CFU/g) were treated as zero. Principal component analysis (PCA) was conducted using the prcomp function on autoscaled data (mean-centred and unit variance). Visualisation was performed with factoextra and ggplot2 packages. Separate PCA models were generated for the overall dataset, viola samples (colour × storage), and snapdragon samples (colour × producer). Pearson correlation coefficients were calculated using the cor function and visualised as an upper-triangular matrix with corrplot, with a scale from −1 to +1. A heatmap of standardised data (Z-scores) with hierarchical clustering (Euclidean distance, Ward’s method) applied to samples was generated using pheatmap.

Results of CFU values were expressed as mean ± standard deviation. Significant differences among data were computed by using ANOVA and the Tukey *t*-test (*p* < 0.05) (XLStat 2012.6.02 statistical pocket, Addinsoft Corp., Paris, France).

## 3. Results and Discussion

### 3.1. Mixed Edible Flowers Microbial Characterisation

There is currently little scientific research on the microbiological safety of edible flowers [11,30], so the first step was a preliminary assessment of the initial microbial population level in marketed mixed flowers and of the microbial increase along refrigerated storage. At time zero, samples from Brands 1 and 2 showed microbial loads below 3 Log CFU/g (Supplementary Figure S1). For Brand 2, this value was consistent with the sampling time, which corresponded to two days after harvest. Despite storage at low temperature, after 5 days, microbial counts increased exponentially, reaching up to 8 Log CFU/g in the Anonymous sample. Even for the sample with the lowest initial microbial population level (2.61 ± 0.14 CFU/g), an increase up to 6 Log CFU/g was observed (Supplementary Figure S1).

To obtain indications about the flower microbiome composition, a further batch of mixed flowers from Brand 2 was analysed by counting on selective media. Brand 2 flowers were preferred and were the only ones among those commercially available with an indication of the harvest/packaging date. Samples were analysed on the day of purchase, actually two days from the harvest date reported on the label, and again after five days of storage at 4 °C. At both sampling points, *Salmonella* and *Listeria* spp. were absent, while both coagulase-positive staphylococci and *Bacillus cereus* were beyond the method’s detection limit.

Although *Salmonella* is the most commonly reported pathogen in recent RASFF reports, evidence of edible flowers playing a role in *Salmonella* transmission dates back to 2010. In that year, *Salmonella* was identified as the cause of an outbreak of salmonellosis after a wedding party in Germany [31].

Even though there is no legal requirement for *Salmonella* content in edible flowers, the absence of this pathogen in 25 g is mandated by current EU legislation (Commission regulation EC, 2007) for fresh-cut fruit and vegetables, sprouts and sprouted seeds, and unpasteurised juices [32]. Results agreed with those reported by Wilczyńska et al. [7] for edible flowers produced in Poland and clearly indicate that appropriate agricultural and hygienic practices were followed during product harvesting and processing.

**Figure 1:**
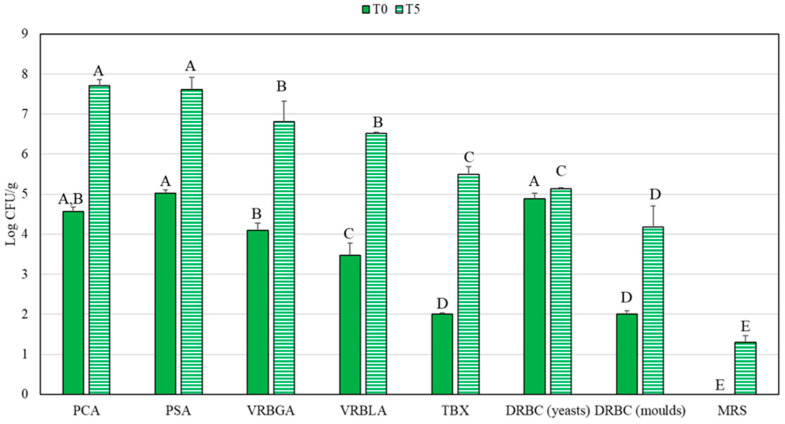
Total microflora (PCA), *Pseudomonadaceae* (PSA), *Enterobacteriaceae* (VRBGA), *Coli-aerogenes* group (VRBLA), *E. coli* (TBX), yeast and moulds (DRBC), and LAB (MRS) counts (Log CFU/g ± SD) in mixed edible flowers from Brand 2 at time zero and after 5 days of storage at 4 °C. Count means with different letters at the same sampling time are statistically significant (*p* < 0.05).

The total microflora on PCA (Figure 1) was more than one Log higher than that recorded in the previous sampling of flowers from the same producer (Supplementary Figure S1 and Figure 1). After cold storage of the same duration, the mesophilic aerobic microflora counted on PCA approached 8 Log CFU/g and mainly consisted of *Pseudomonadaceae*, as confirmed by the comparable count levels obtained on PSA medium (Figure 1). The level of *Enterobacteriaceae* on VRBGA was one Log lower than that recorded on PSA. In this case, the overlap of VRBGA/VRBLA counts also apparently suggests that *Enterobacteriaceae* were almost entirely represented by bacteria belonging to the so-called *Coli-aerogenes* group, more commonly reported as coliforms (Figure 1). Regarding *Escherichia coli*, although the count levels on TBX medium were quite high, none of the colonies displayed the typical appearance. Indeed, this substrate is known to support the growth of coliforms other than *E. coli* [33].

LAB on MRS, initially absent, increased slightly above one Log after cold storage. Being oxygen-tolerant anaerobes, LAB might find favourable conditions for growth once the aerobic microflora and the plant tissue itself have consumed the oxygen in the package headspace through respiration.

Population levels on DRBC were around 5 Log CFU/g and mainly consisted of yeasts. Moulds, selectively counted, were about three Logs lower at T0 and roughly one Log lower after cold storage (Figure 1).

In general, a five-day refrigerated storage period led to unacceptable microbial growth. Furthermore, this storage length is hardly consistent with the necessary aesthetic appeal of decorative edible flowers. From this perspective, the application of preservation techniques such as modified atmosphere packaging or UV treatments should be assessed.

### 3.2. Violet and Pansy Microflora

**Figure 2:**
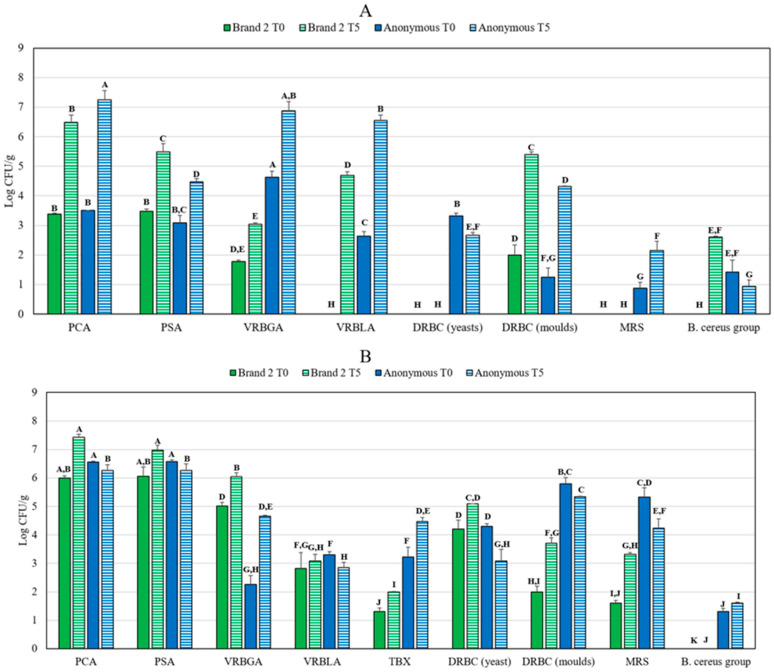
Total microflora (PCA), *Pseudomonadaceae* (PSA), *Enterobacteriaceae* (VRBGA), *Coli-aerogenes* group (VRBLA), *E. coli* (TBX), yeast and moulds (DRBC), LAB (MRS), and *B. cereus* group counts (Log CFU/g ± SD) in pansies (Panel (**A**)) and snapdragon (Panel (**B**)) from Brand 2 and Anonymous at time zero and after 5 days of storage at 4 °C. Count means with different letters at the same sampling time are statistically significant (*p* < 0.05).

A microbiological characterisation was also carried out on two batches of pansies produced by Brand 2 and Anonymous. Indeed, the Brand 2 trays contained both violets (*Viola cornuta*) and pansies (*Viola* × *wittrockiana*), two taxonomically distinct species (Supplementary Figure S2). In none of the analysed samples were coagulase-positive staphylococci or *E. coli* detected (<10 CFU/g). *Salmonella* and *Listeria* spp. were also absent In both pansy samples, *Pseudomonadaceae* on PSA did not seem to be the dominant bacteria. Flowers reported as Anonymous, and therefore without certainty about the harvest date, appeared largely contaminated with *Enterobacteriaceae* (VRBGA), with contamination increasing to around 7 Log CFU/g. At the same time lapse, bacteria from the *Coli-aerogenes* group (VRBLA) increased to almost the same level (Figure 2A).

Results did not match those reported by Fernandes and coworkers [34] for uncoated white pansies: PCA counts were higher (4.83 ± 0.73 Log CFU/g), even though flowers were analysed immediately after harvest. Additionally, after two weeks of storage, the microbial increase was less than 1 Log, whereas in the present survey, flowers stored under the same conditions experienced at least a 3 Log increase. Coliforms, which became dominant during storage in both pansy batches, dropped below the method’s threshold in the study conducted by Fernandes et al. [34]. Actually, on pansies collected at the same site, the same authors reported even higher PCA counts in a previous study [35]; PCA counts at time zero were over 7 Log CFU/g, while coliforms were just around one Log CFU/g [35].

In both samples (Brand 2 and Anonymous), a moderate presence (below two Logs) of presumptive *B. cereus* was detected for the first time. The so-called *B. cereus* group consists of eight closely related species: *B. cereus*, *B. thuringiensis*, *B. pseudomycoides*, *B. anthracis*, *B. mycoides*, *B. weihenstephanensis*, *B. cytotoxicus*, and *B. toyonensis*, among which most are of economic and clinical relevance [36].

*B. cereus* and *B. thuringiensis* are frequently detected in foods since they are largely present in agricultural soil. *B. cereus* is a second risk priority group of foodborne illnesses in fresh agricultural products, and its contamination is one of the major problems in vegetables [37]. On the other hand, *B. thuringiensis* (Bt)-based plant protection products are among the most widely used biopesticides worldwide [38]. Since these two bacteria cannot be discriminated using plate count methods [39], the occurrence of *B. thuringiensis* spores used as a pesticide cannot be ruled out. Actually, the persistence of *B. thuringiensis* spores in the final product, coupled with the problematic discrimination *B. thuringiensis*/*cereus*, led to several controversial circumstances [39].

A total of 30 colonies were isolated from PSA, PCA, and MRS counting plates of the Brand 2 samples. Cultures were purified and identified by 16S rDNA sequencing (Supplementary Table S1). Six strains from PSA could be reported to *Pseudomonas parafulva* with a percent of similarity in the range 99.62–100%. The PSA medium also allowed the detection of one *P. juntendi* (100% similarity with NR_180497.1) and one *P. punonensis* (99.72% with NR_025385.1). The presence of *Pseudomonas* species is not surprising, since members of this genus are largely represented in the microbiota of vegetables and Ready-to-Eat vegetable salads [40]. Anyhow, the species *Pseudomonas juntendi* is a newly recognised opportunistic pathogen that often exhibits multidrug resistance and is commonly isolated from clinical settings [41].

Apart from the *Pseudomonas* genus, cultures isolated from PSA could be reported to *Enterobacter asburiae* (two strains, 99.17 and 99.41% with NR_024640.1), *Delftia lacustris* (100% with NR_116495.1), and *Winslowiella iniecta* (99.73% with NR_112880.1).

Five isolates from PCA could be identified as *Ralstonia mannitolilytica* (99.19–100%) and five as *Pantoea agglomerans* (n. 3 strains 99.50–99.53% with NR_041978.1; n. 2 99.39–99.68% with NR_114111.1). Both species are regarded as plant pathogens, once thought to be restricted to the plant kingdom, that can also behave as opportunistic pathogens in humans, particularly those who are immunocompromised [42,43].

Cultures from MRS were not LAB. By 16S rDNA sequencing, two strains could be equally reported to both *Asaia spathodeae* (NR_112953.1) and *Asaia prunellae* (NR_112880.1).

Indeed, many of the species detected have been associated with human diseases. *Enterobacter* species [44] and *Acinetobacter baumannii* [45], for instance, are both included among the “ESKAPE” pathogens, i.e., as key causes of healthcare-associated infections that are often multidrug-resistant.

In general, the results did not match those reported by Wetzel et al. [46] for mixed flower samples of *Viola tricolor* var. *hortensis*, *Pelargonium*, and *Tropaeolum* spp. According to the authors, the most represented genus was *Enterobacter* spp. Such a discrepancy might be related to the different methodological approach. In the study by Wetzel and coworkers, samples underwent an overnight enrichment phase in Brain Heart Infusion medium at 37 °C [46]. This incubation likely shifted the microbial balance in favour of genera better adapted to mesophilic conditions.

### 3.3. Snapdragon Microflora

Two batches of snapdragons (Brand 2 and Anonymous) were analysed (Figure 2B). In both cases, the initial microbial loads on PCA were clearly higher than those recorded for pansies, and the dominant microflora appeared to be *Pseudomonadaceae*, as evidenced by the near-perfect overlap of PSA and PCA count values. Furthermore, a noticeable difference emerged from the data comparison. In the Anonymous samples, LAB on MRS and moulds on DRBC dominated, whereas in samples from Brand 2, *Enterobacteriaceae* on VRBGA and yeast on DRBC exhibited higher counts (Figure 2B). Despite being rather high, especially after cold storage, TBX counts did not indicate the presence of *E. coli*, since none of the colonies exhibited the typical aspect. Analogously, the microscope observation of randomly chosen colonies grown on MRS revealed the presence of yeasts and moulds even on plates seeded with the highest dilutions.

Forty-four cultures were identified from PCA, PSA, and MRS plates of the Brand 2 counting plates at time zero. Twenty strains (more than 43% of the isolates) could be reported to the species *Rosenbergiella epipactidis* (Supplementary Table S2). This species was isolated from plates seeded with the 10^−7^ dilution, and the identification rate was always 99–100%. The genus *Rosenbergiella* (order *Enterobacterales*, class *Gammaproteobacteria*) is one of the most frequent bacterial inhabitants of flowers from phylogenetically diverse plants worldwide, and it is also frequently isolated from insects and other flower visitors [47]. In recent years, there has been growing interest in studying members of this genus due to their ability to withstand the harsh conditions of floral nectar, which typically include high osmotic pressure, low nitrogen availability, and plant-derived toxins [48]. Furthermore, the bacterial community of edible red mini roses was dominated by this species [49].

Pseudomonads were also quite represented, with 8 strains out of 44. Apart from the species *P. oryzihabitans* (n. 3 strains), *P. putida* (n. 1), and *P. boreofloridensis* (n. 1), it is noteworthy that three cultures were reported as *P. aeruginosa* (Supplementary Table S2), an opportunistic human pathogen causing nosocomial infections [50]. A recent study published in The Lancet indicated that *P*. *aeruginosa*, alongside four other pathogens (*Staphylococcus aureus*, *E. coli*, *Streptococcus pneumoniae*, and *Klebsiella pneumoniae*), accounts for 54.9% of bacterial-associated fatalities [51].

### 3.4. Comparison of Viola and Snapdragon Microflora

**Figure 3:**
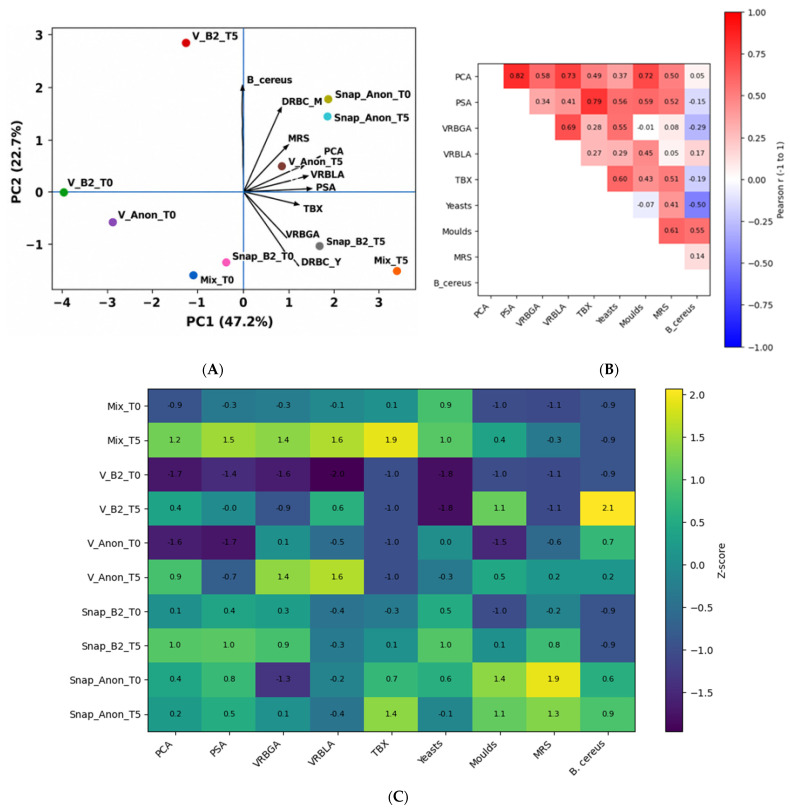
Multivariate analysis of microbial counts (Log CFU/g) in edible flowers (M: mixed; V: violets; Snap: snapdragon) at T0 and T5 from the Brand 2 (B2) and Anonymous (Anon) producers. (**A**) PCA biplot showing sample distribution and variable loadings. (**B**) Pearson correlation matrix with a colour scale ranging from 1 (negative correlation, blue) to +1 (positive correlation, red). (**C**) Heatmap of standardised microbial counts (Log CFU/g) in edible flower samples. Values are shown as Z-scores to highlight relative differences among samples and microbial groups.

To better understand the structure, variability, and interrelationships of microbial communities in edible flowers, multivariate analyses (principal component analysis, hierarchical clustering, and correlation analysis) were employed. Principal component analysis (Figure 3A) revealed that storage time was the primary factor shaping microbial composition. The first principal component (PC1), explaining 47.2% of the total variance, clearly separated fresh (T0) from stored (T5) samples, reflecting a general increase in microbial load during refrigerated storage. This shift was mainly driven by total aerobic microflora (PCA), *Pseudomonadaceae* (PSA), *Enterobacteriaceae* (VRBGA), and coliforms (VRBLA), which showed strong positive loadings along PC1. The second principal component (PC2), accounting for 22.7% of the variance, differentiated samples according to flower type and producer, highlighting variability in specific microbial groups such as moulds, yeasts, LAB (MRS), and the *B. cereus* group.

These patterns were further supported by the hierarchical clustering of samples, which revealed distinct groupings consistent with principal component analysis results. Fresh viola samples clustered together and were characterised by low microbial loads, indicating relatively high microbiological quality at the point of purchase. In contrast, samples stored for 5 days formed separate clusters associated with increased levels of multiple microbial groups, confirming the strong effect of storage on microbial proliferation. Snapdragon samples, particularly those from the Anonymous producer, formed a distinct cluster characterised by higher levels of moulds, yeasts, and LAB, suggesting a specific microbial ecology linked to the production source. Viola samples at T5 showed a distinct shift associated with increased moulds and *B. cereus*, indicating flower-type-specific postharvest dynamics (Figure 3A).

Pearson correlation analysis provided further insight into relationships among microbial groups and supported the interpretation of the principal component analysis (Figure 3B). Strong positive correlations were observed between total aerobic microflora (PCA) and *Pseudomonadaceae* (PSA) (r = 0.82), as well as with coliforms (VRBLA) (r = 0.73) and moulds (r = 0.72), indicating that microbial proliferation during storage involves coordinated increases across multiple microbial groups. Similarly, PSA showed a strong correlation with TBX (r = 0.79), suggesting parallel growth of spoilage-associated bacteria.

In contrast, some microbial groups exhibited weaker or distinct relationships. *Enterobacteriaceae* (VRBGA) showed only moderate correlations with total counts and weak or negative associations with moulds and *B. cereus*, indicating a partially independent behaviour. Notably, the *B. cereus* group showed weak or negative correlations with most microbial groups, suggesting that its occurrence is likely driven by specific contamination events rather than general microbial growth. Moulds and LAB (MRS) displayed a moderate positive correlation (r = 0.61), supporting their co-occurrence in specific samples, particularly snapdragon, and contributing to separation along PC2 in the principal component analysis (Figure 3B).

Overall, the combined multivariate approach demonstrates that storage is the dominant driver of microbial dynamics in edible flowers, leading to a coordinated increase in several bacterial groups, while specific microbial populations such as moulds, LAB, and *B. cereus* follow distinct, sample-dependent patterns influenced by flower type and production source. These findings highlight the importance of considering both general microbial proliferation and targeted microbial risks when assessing the microbiological safety of edible flowers intended for raw consumption.

A heatmap based on standardised microbial counts highlighted clear differences among samples and microbial groups (Figure 3C). Samples stored for 5 days generally showed higher relative levels of total aerobic microflora, *Pseudomonadaceae*, *Enterobacteriaceae*, coliforms, and TBX counts than the corresponding T0 samples, confirming the strong effect of refrigerated storage on microbial proliferation. Among the analysed samples, Brand 2 violas at T0 showed the lowest overall microbial profile, whereas mixed flowers at T5 and the snapdragon samples displayed higher relative abundance across several microbial groups. Snapdragon samples from the Anonymous producer were particularly associated with elevated mould and MRS values, whereas Brand 2 pansies at T5 were characterised by a marked increase in mould and the *B. cereus* group. Overall, the heatmap supports the PCA results by showing that storage time was the main factor shaping microbial patterns, with additional variability associated with flower type and producer.

To conclude, while the impact of storage—even when refrigerated—on microbial contamination levels is to be expected, the effect of flower morphology is an issue here investigated for the first time. Although further confirmation is required, the data gathered in this study suggest that violets, rather than snapdragons, pose a lower microbiological risk. This effect may be attributable to the corolla’s less upright structure. Due to the shape, the corolla may be more exposed to the sterilising effects of sunlight and less vulnerable to waterlogging.

### 3.5. Flower Microflora Based on the Corolla Colour

Pansies from Brand 2 were split by colour and separately analysed at both T0 and T5 (Table 1). Pansy microflora was found to be dominated by *Enterobacteriaceae* rather than *Pseudomonadaceae*, although some differences emerged. Specifically, counts on PCA, PSA, and VRBGA were statistically higher in deep purple and orange/violet flowers (Table 1).

Flowers are important for many insects, most notably because they provide food. Flower colours are thought to be tuned to the visual systems of pollinators and have largely evolved to attract pollinators [52]. In addition to a primary preference for blue, a preference for orange to red and purple (a mixture of red and blue) has been recorded in many butterfly species [53]. Flower colour and animal colour vision are complex issues, and colour should not be considered a ‘trait’ but rather is a perception. Nevertheless, the optical properties of flowers have been proven to affect the visual signalling between flowers and pollinators [54].Also, two batches (Brand 2 and Anonymous) of snapdragon were analysed after being split by colour (Table 2). As a general consideration, the Anonymous flowers appeared to be characterised by higher microbial loads, especially with reference to total aerobic microflora on PCA, *Pseudomonadaceae* on PSA, *Enterobacteriaceae* on VRBGA, and moulds on DRBC. Furthermore, LAB on MRS were only retrieved in this sample, whereas they remained below the detection limit in flowers from Brand 2 (Table 2). Indeed, both samples were analysed on the day of purchase, which was two days after the date label-stated for Brand 2. Conversely, it was impossible to speculate about how long the Anonymous samples had been stored before the examination.

In both cases, regarding the corolla colour, more than one significant difference emerged. In Brand 2 flowers, the amber snapdragon appeared more contaminated, especially with respect to population levels recorded on PCA, PSA, VRBGA, yeasts, and moulds. Conversely, flowers of the red-orange colour appeared less contaminated (Table 2). Snapdragon corollas of the Anonymous brand were of different colours, so an exact parallelism between the two datasets could not be traced (Table 2). To some extent, also in such a case, different colours were characterised by different microbial levels, especially in terms of total microflora on PCA, *Pseudomonadaceae* on PSA, *Enterobacteriaceae* on VRBGA, and LAB on MRS. Specifically, vermilion was the snapdragon colour with the highest microbial levels, while fuchsia was the least contaminated (Table 2).

From this perspective, different microbial population levels in flowers of a given colour might, to some extent, reflect variation in attractiveness to pollinating insects. In the snapdragon case, it could be the yellow tone known as amber. Indeed, yellow artificial flowers were the preference exhibited by *Bombus terrestris* and *Lasioglossum villosulum* [55], as well as for the hoverfly *Episyrphus balteatus* [56].

**Figure 4:**
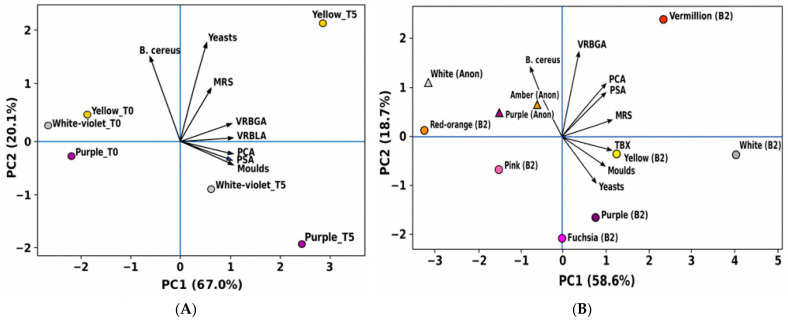
Principal component analysis (PCA) of microbial counts (Log CFU/g) in colour-defined edible flower samples. (**A**) Viola flowers analysed at T0 and T5. (**B**) Snapdragon flowers analysed at T0. Arrows represent variable loadings. Samples are coloured according to corolla colour and labelled according to producer (B2 = Brand 2; Anon = Anonymous), with circles representing Brand 2 and triangles representing the Anonymous samples.

In addition, principal component analysis was used to evaluate the influence of storage time, flower colour, and producer on the microbial composition of edible flowers (Figure 4). Separate models were constructed for viola and snapdragon samples to account for the unbalanced experimental design, as storage data were available only for violas.

In violas, the analysis revealed that storage time was the dominant factor influencing microbial composition. The first principal component (PC1), explaining the majority of variance, clearly separated fresh (T0) from stored (T5) samples, indicating a consistent increase in microbial load during storage. This shift was primarily associated with total aerobic microflora (PCA), *Pseudomonadaceae* (PSA), *Enterobacteriaceae* (VRBGA), and coliforms (VRBLA), reflecting general microbial proliferation. Although colour-related differences were less pronounced at T0, they became more evident after storage, suggesting that flower-specific traits may influence microbial growth dynamics. Yellow flowers showed the strongest divergence, associated with higher levels of yeasts, moulds, and LAB, indicating increased susceptibility to microbial development during storage (Figure 4A).

In snapdragon, where only T0 samples were analysed, PCA revealed colour-dependent variability in microbial composition already present on the day of purchase. Certain colours, particularly white (Brand 2) and vermilion, displayed distinct microbial profiles, while other colours formed more compact clusters. In addition to colour effects, a partial separation between the Brand 2 and Anonymous samples was observed, indicating that production and postharvest handling conditions contribute to microbial variability. This suggests that both intrinsic factors (such as floral morphology and colour) and extrinsic factors (such as cultivation and handling practices) shape the initial microbiological status of edible flowers (Figure 4B).

Overall, the combined principal component analyses demonstrate that storage is the primary driver of microbial changes in edible flowers, leading to a coordinated increase in several microbial groups. In contrast, flower colour and producer contribute to secondary variability, influencing both the initial microbial composition and the extent of microbial proliferation during storage. These findings highlight the importance of considering both product origin and postharvest conditions when assessing the microbiological safety of edible flowers intended for raw consumption.

### 3.6. Edible Flowers Microbiome by HTS

**Figure 5:**
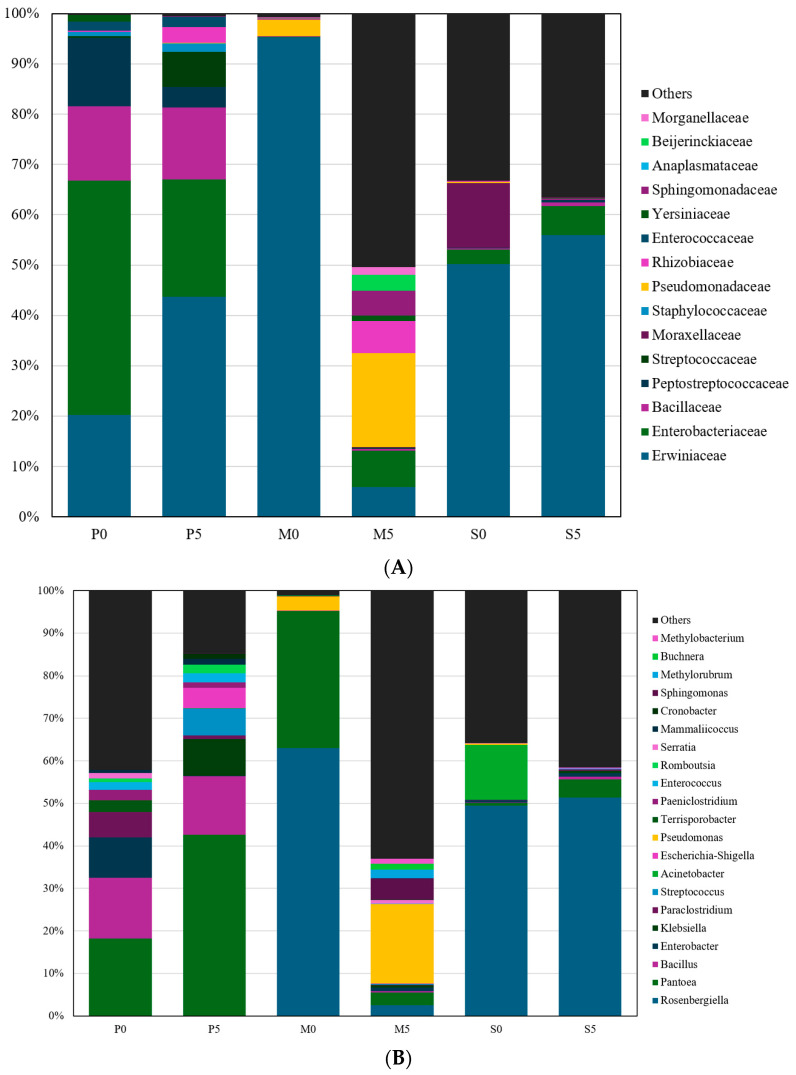
Bar plots showing the mean relative abundance of bacterial family (Panel (**A**)) and genera (Panel (**B**)) in pansy (P0 and P5), mixed (M0 and M5), and snapdragon (S0 and S5) flowers at time zero and after 5 days of storage. Only taxa with a mean relative abundance > 1% are plotted.

The bacterial community of mixed flowers (M0 and M5), pansy (P0 and P5), and snapdragon (S0 and S5) from Brand 2 was evaluated (Figure 5).

Results for mixed flowers (samples M0 and M5) did not reflect those obtained by counting on selective media (Figure 1). In fact, at time zero, the majority of the ASV reads could be reported to the *Erwiniaceae* family (Figure 5A), and at the genus level, the most represented were *Rosenbergiella* and *Pantoea* spp. (Figure 5B). After cold storage (M5), the relative abundance of the genus *Pseudomonas* spp. increased, confirming results reported by conventional counting methods. Conversely, the expected increase in coliforms could not be inferred: only the abundance in the ‘Others’ undefined taxa increased (Figure 5B).

In pansies (P0 and P5), the HTS application fully reflected the microbial characterisation obtained by counts on selective media: *Enterobacteriaceae* and *Bacillaceae* were the most represented families (Figure 5A). Conversely, *Enterobacteriaceae* seemed to decrease during cold storage, while the genus *Pantoea* spp. (Figure 5B) within *Erwiniaceae* increased (Figure 5A). In snapdragon, the genus *Rosenbergiella* spp. dominated (Figure 5B), while *Moraxellaceae*, and specifically the genus *Acinetobacter* spp., decreased (Figure 5). Indeed, by culture isolation, one strain of *Acinetobacter schindleri* was isolated (Supplementary Table S2).

*Bacillaceae* were not detected; despite the cultures’ identification, two strains could be reported to spore-forming genera such as *Bacillus* and *Solibacillus* spp. (Supplementary Table S2).

## 4. Conclusions

Despite their appealing and seemingly pristine appearance, edible flowers may serve as a vehicle for a range of microbial, chemical, and environmental contaminants. In all the analysed samples, microbial populations were definitely not negligible, and the increase during cold storage was consistently substantial. Although primary pathogens were never detected, numerous opportunistic pathogens were isolated. Challenges to public health and the economy on national and local scales posed by vegetables [57] are ineludibly heightened in the case of edible ornamental flowers. Flowers meant to be used as garlands for dishes and drinks are never cooked and hardly even washed. Additionally, flowers are often used to decorate cakes and pastries, namely foods that may have a rather lengthy shelf life.

The difference in microbial community seems to be linked to the flower shape and colour. To identify cultivars that are less susceptible to microbial colonisation, this issue should be thoroughly investigated.

However, to substantiate potential public health implications, further investigations are required, including dose–response and exposure assessments, illness data, virulence confirmation, and antimicrobial resistance testing. Nevertheless, based on these preliminary findings, producers should be made aware of the importance of using sound agricultural and hygiene methods to produce and sell contamination-free edible flowers.

The need for clear and detailed labels should also be instilled. Promoting responsible consumption will, in fact, depend on the public having access to reliable, accurate, and consistent information about the handling, safety, and appropriate usage of edible flowers.

## Figures and Tables

**Table 1:** Microbial groups evaluated on selective media as described in the Figure 2 caption for three varieties of pansy flowers from Brand 2 at time zero and after 5 days of storage at 4 °C. For data with different letters, microbial count differences expressed as Log CFU/g ± SD are statistically significant (*p* < 0.05).

Media	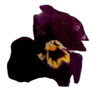 Deep Purple		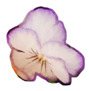 White Edged in Violet		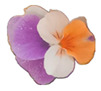 Yellow	
	T0	T5	T0	T5	T0	T5
PCA	4.20 ± 0.13 _b_	7.97 ± 0.06 _a_	2.75 ± 0.19 _c_	7.56 ± 0.32 _a_	3.82 ± 0.24 _b_	8.15 ± 0.23 _a_
PSA	3.40 ± 0.30 _b_	4.48 ± 0.28 _a_	2.60 ± 0.21 _b_	4.48 ± 0.10 _a_	3.26 ± 0.24 _b_	4.48 ± 0.06 _a_
VRBGA	4.48 ± 0.30 _c_	7.07 ± 0.21 _a_	3.92 ± 0.24 _c_	5.78 ± 0.16 _b_	5.48 ± 0.12 _b_	7.78 ± 0.19 _a_
VRBLA	4.04 ± 0.14 _c_	6.82 ± 0.33 _a_	3.85 ± 0.22 _c_	5.60 ± 0.15 _b_	4.00 ± 0.30 _c_	7.20 ± 0.30 _a_
DRBC (yeasts)	3.13 ± 0.18 _b_	3.00 ± 0.16 _b_	3.17 ± 0.23 _b_	3.20 ± 0.28 _b_	3.68 ± 0.33 _b_	5.00 ± 0.42 _a_
DRBC (moulds)	1.36 ± 0.15 _c_	4.82 ± 0.28 _a_	1.36 ± 0.11 _c_	3.85 ± 0.20 _b_	1.00 ± 1.41 _c_	4.30 ± 0.17 _a,b_
MRS	0.00 ± 0.00 _c_	2.30 ± 0.32 _b_	2.60 ± 0.07 _b_	0.00 ± 0.00 _c_	0.00 ± 0.00 _c_	4.16 ± 0.23 _a_
*B. cereus* group	1.48 ± 0.13 _a_	0.00 ± 0.00 _b_	1.30 ± 0.25 _a_	1.30 ± 0.11 _a_	1.48 ± 0.38 _a_	1.48 ± 0.13 _a_

**Table 2:** Microbial groups evaluated at time zero for different snapdragon varieties from Brand 2 (red orange, pink, white, purple, and amber) and Anonymous (fuchsia, yellow, deep purple, white, and vermilion). For data with different letters, microbial count differences expressed as Log CFU/g ± SD are statistically significant (*p* < 0.05).

	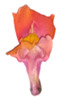 Red Orange	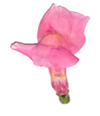 Pink	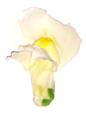 White	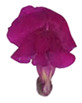 Purple	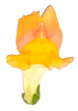 Amber	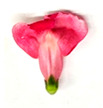 Fuchsia	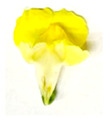 Yellow	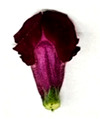 Deep Purple	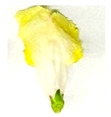 White	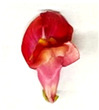 Vermilion
PCA	4.52 ± 0.28 _f_	5.03 ± 0.16 _ef_	5.07 ± 0.16 _def_	5.62 ± 0.23 _cde_	6.07 ± 0.21 _cd_	4.99 ± 0.32 _ef_	5.94 ± 0.10 _cde_	6.13 ± 0.38 _bc_	7.13 ± 0.19 _ab_	7.74 ± 0.36 _a_
PSA	4.22 ± 0.13 _d_	4.82 ± 0.23 _bcd_	4.77 ± 0.25 _cd_	5.26 ± 0.21 _bc_	5.22 ± 0.11 _bc_	4.86 ± 0.17 _bcd_	5.40 ± 0.21 _bc_	5.69 ± 0.19 _b_	7.10 ± 0.30 _a_	7.45 ± 0.32 _a_
VRBGA	4.00 ± 0.21 _c_	3.99 ± 0.32 _c_	5.02 ± 0.19 _bc_	5.02 ± 0.28 _bc_	5.40 ± 0.25 _b_	2.74 ± 0.27 _d_	5.04 ± 0.45 _bc_	2.04 ± 0.27 _d_	5.72 ± 0.22 _b_	6.86 ± 0.17 _a_
VRBLA	1.50 ± 0.40 _de_	2.82 ± 0.24 _c_	1.30 ± 0.20 _e_	3.00 ± 0.57 _c_	2.82 ± 0.27 _c_	2.60 ± 0.22 _cd_	4.36 ± 0.15 _b_	2.11 ± 0.42 _cde_	5.57 ± 0.25 _a_	2.00 ± 0.42 _cde_
TBX	0.00 ± 0.00 _e_	2.92 ± 0.10 _bc_	0.00 ± 0.00 _e_	0.00 ± 0.00 _e_	2.92 ± 0.36 _bc_	2.73 ± 0.38 _c_	3.73 ± 0.06 _b_	1.78 ± 0.28 _d_	5.75 ± 0.28 _a_	3.15 ± 0.23 _bc_
DRBC (yeasts)	2.92 ± 0.29 _c_	3.88 ± 0.20 _abc_	3.22 ± 0.16 _bc_	3.70 ± 0.30 _abc_	4.26 ± 0.42 _ab_	3.90 ± 0.28 _abc_	4.00 ± 0.25 _abc_	4.00 ± 0.28 _abc_	4.72 ± 0.19 _a_	3.30 ± 0.34 _bc_
DRBC (moulds)	3.40 ± 0.21 _b_	2.92 ± 0.23 _b_	0.00 ± 0.00 _c_	2.92 ± 0.57 _b_	3.52 ± 0.11 _b_	4.97 ± 0.51 _a_	5.22 ± 0.29 _a_	6.07 ± 0.26 _a_	5.68 ± 0.05 _a_	6.03 ± 0.35 _a_
MRS	0.00 ± 0.00 _d_	0.00 ± 0.00 _d_	0.00 ± 0.00 _d_	0.00 ± 0.00 _d_	0.00 ± 0.00 _d_	2.45 ± 0.33 _c_	2.60 ± 0.21 _c_	3.56 ± 0.32 _b_	5.26 ± 0.26 _a_	5.85 ± 0.18 _a_
*B. cereus* group	1.90 ± 0.16 _a_	1.78 ± 0.30 _a_	1.78 ± 0.16 _a_	1.60 ± 0.40 _a_	2.08 ± 0.11 _a_	1.00 ± 0.42 _a_	1.50 ± 0.42 _a_	1.10 ± 0.26 _a_	0.90 ± 0.30 _a_	1.50 ± 0.31 _a_

## Data Availability

The original contributions presented in this study are included in the article/Supplementary Materials. Further inquiries can be directed to the corresponding author.

## Supplementary Materials

The following supporting information can be downloaded at https://www.mdpi.com/article/10.3390/foods15152697/s1, Figure S1: Mesophilic aerobic microflora expressed as CFU/g (mean ± SD) in packaged edible ornamental flowers marketed in the province of Naples (Campania Region, Italy) by three producers (Brand 1, Brand 2, and Anonymous) on the day of purchase and after 5 days at 4 °C. Labels were obscured to ensure privacy; Figure S2: Packaged edible pansies—*Viola cornuta* and *Viola* × *wittrockiana*—analysed. Labels were obscured to ensure privacy; Table S1: Results from comparing the 16S rRNA sequences of selected pansy isolates with the Bacterial 16S Ribosomal RNA Database (NCBI). Only the closest relatives obtained in each case are shown; Table S2: Results from comparing the 16S rRNA sequences of selected snapdragon isolates with the Bacterial 16S Ribosomal RNA Database (NCBI). Only the closest relatives obtained in each case are shown.
