# Supplementary material for: Microbiological Safety of Ornamental Edible Flowers
Source: Foods. 2026 Jul 30;15(15):2697. doi: 10.3390/foods15152697 (PMC13464742; doi:10.3390/foods15152697)
Supplement: Supplementary file 1 [file foods-15-02697-s001.zip › foods-4411486-supplementary.pdf]

Supplementary Figure S1. Mesophilic aerobic microflora expressed as CFU/g (mean ± SD) in packaged edible ornamental flowers marketed in the province of Naples (Campania Region, Italy) by tree producers (Brand 1, Brand 2, and Anonymous) on the day of purchase and after 5 days at 4°C. For data with different letters, microbial count differences are statistically significant (p < 0.05). Labels were obscured to ensure privacy

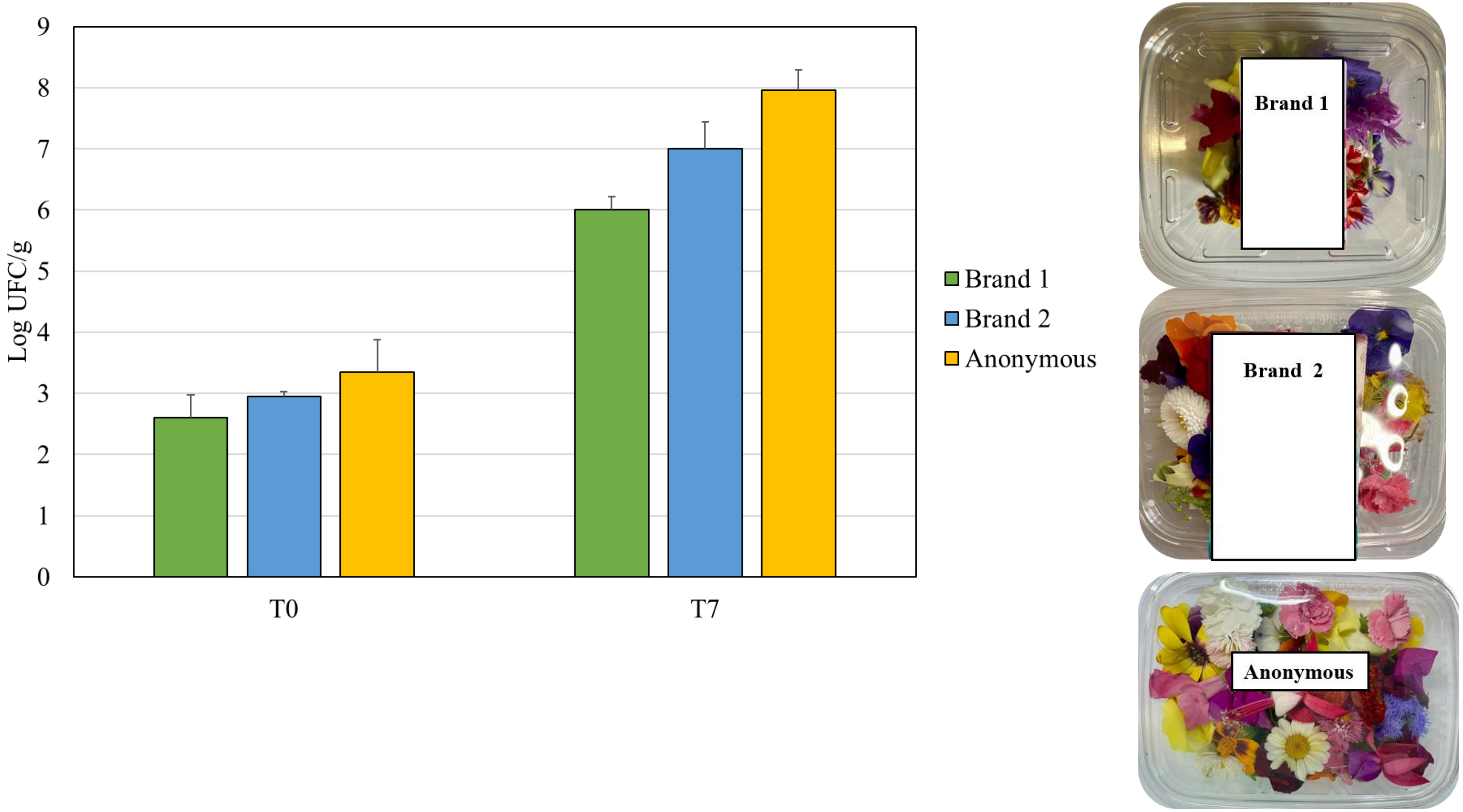

Supplementary Figure S2. Packaged edible pansies – *Viola cornuta* and *Viola* × *wittrockiana* - analysed. Labels were obscured to ensure privacy

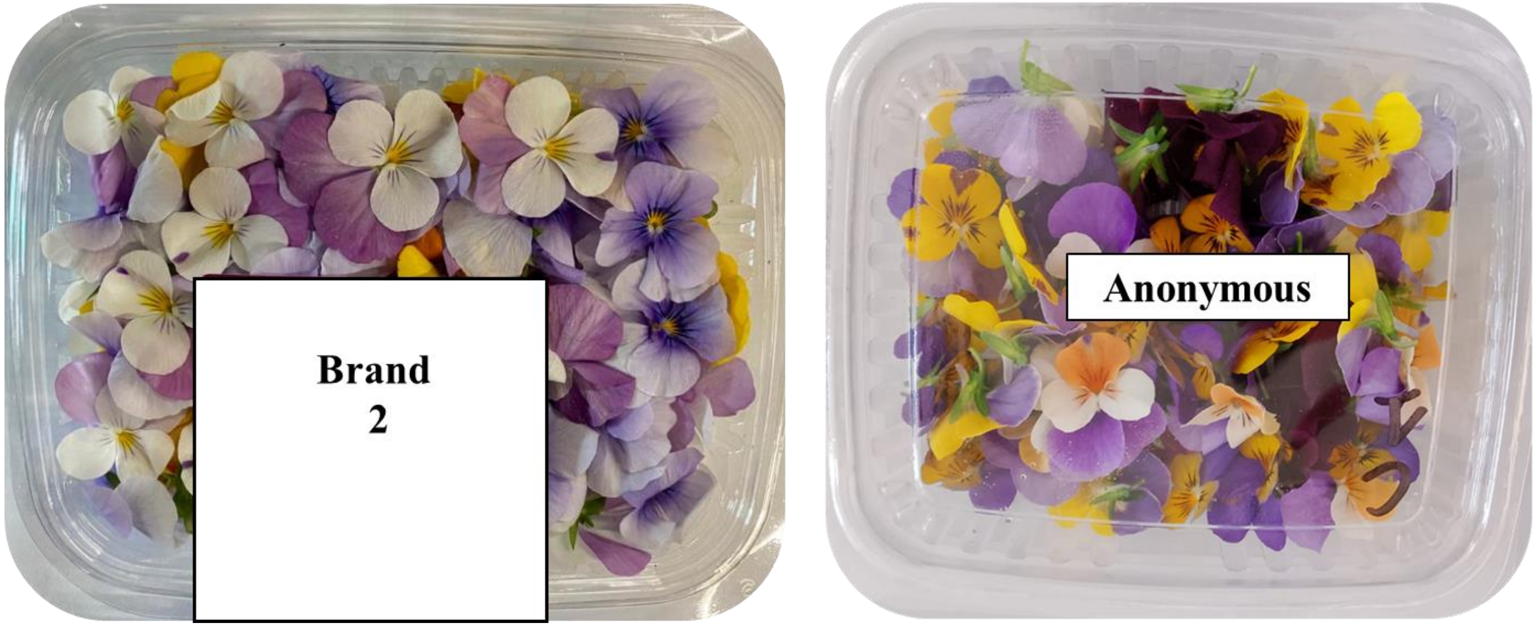

Supplementary Table S1. Results from comparing the 16S rRNA sequences of selected pansy isolates with the Bacterial 16S Ribosomal RNA Database (NCBI). Only the closest relatives obtained in each case are shown.

Medium	N. strains	Taxon	% Ident	Accession
PCA	3	*Pantoea agglomerans*	99.50-99.53%	NR_041978.1
	2	*Pantoea agglomerans*	99.39-99.68%	NR_114111.1
	5	*Ralstonia mannitolilytica*	99.19-100.00%	NR_025385.1
	3	*Cupriavidus cauae*	99.71-100.00%	NR_180770.1
	1	*Agrobacterium fabrum*	99.72%	NR_074266.1
	1	*Acinetobacter baumannii*	99.71%	NR_113237.1
	1	*Sphingomonas changnyeongensis*	98.00%	NR_180866.1
PSA	5	*Pseudomonas parafulva*	99.72-99.91%	NR_113856.1
	1	*Pseudomonas parafulva*	100.00%	NR_040859.1
	1	*Pseudomonas punonensis*	99.72%	NR_109583.1
	1	*Pseudomonas juntendi*	100.00%	NR_180497.1
	1	*Enterobacter bugandensis/asburiae*	99.17%	NR_148649.1/ NR_024640.1
	1	*Enterobacter asburiae*	99.41%	NR_024640.1
	1	*Winslowiella iniecta*	99.61%	NR_137333.1
	1	*Delftia lacustris*	100.00%	NR_116495.1
MRS	1	*Asaia spathodeae/Asaia prunellae*	99.75%	NR_112953.1/ NR_112880.1
	1	*Asaia spathodeae/Asaia prunellae*	99.73%	NR_112953.1/ NR_112880.1

Supplementary Table S2. Results from comparing the 16S rRNA sequences of selected snapdragon isolates with the Bacterial 16S Ribosomal RNA Database (NCBI). Only the closest relatives obtained in each case are shown.

Medium	N. strains	Scientific Name	% Ident	Accession
PCA	9	*Rosenbergiella epipactidis*	99.12-100.00%	NR_126303.1
	3	*Staph. hominis* subsp. *novobiosepticus*	99.81-100.00%	NR_041323.1
	1	*Pantoea ananatis*	98.96%	NR_026045.1
	1	*Leclercia pneumoniae*	99.48%	NR_181872.1
	1	*Pseudomonas putida*	99.87%	NR_114794.1
	1	*Serratia marcescens*	99.62%	NR_114043.1
	1	*Acinetobacter schindleri*	99.66%	NR_025412.1
	1	*Bacillus licheniformis*	87.87%	NR_118996.1
	1	*Solibacillus silvestris*	99.89%	NR_028865.1
PSA	10	*Rosenbergiella epipactidis*	99.74-100%	NR_126303.1
	3	*Pseudomonas aeruginosa*	99.81-100.00%	NR_117678.1
	1	*Pseudomonas oryzihabitans*	99.91%	NR_115005.1
	2	*Pseudomonas oryzihabitans*	99.57-100.00%	NR_114041.1
	1	*Pseudomonas boreofloridensis*	99.42%	NR_199512.1
MRS	4	*Enterococcus hulanensis*	99.87-99.91%	NR_174228.1
	2	*Enterococcus raffinosus*	99.90-100%	NR_026499.1
	2	*Enterococcus faecalis*	99.90-99.91%	NR_113901.1
